# A Family Report of Hereditary Gingival Fibromatosis

**DOI:** 10.1155/2024/2851229

**Published:** 2024-10-16

**Authors:** Mohammed Taib Fatih, Renaz Sabir Saleh, Mohammed Khalid Mahmood, Zana Fuad Noori, Handren Ameer Kurda, Mohammed Aso Abdulghafor

**Affiliations:** ^1^Dentistry Department, Komar University of Science and Technology, Sulaymaniyah, Iraq; ^2^Aix-Marseille University, CNRS, EFS, ADES, Marseille, France; ^3^Dentistry College, American University of Iraq-Sulaimani (AUIS), Sulaymaniyah, Kurdistan, Iraq; ^4^Dentistry College, Sulaimani University, Sulaymaniyah, Kurdistan, Iraq

**Keywords:** gingival fibromatosis, gingival overgrowth, gingivectomy, hereditary gingival fibromatosis

## Abstract

**Background:** Hereditary gingival fibromatosis (HGF) is a rare hereditary condition characterized by abnormal enlargement of the gingival tissue with a variable clinical manifestation. Typically, the hyperplastic gingiva is normal in color and consistency, and the tendency of bleeding is minimal. The swelling may be limited to a particular location or generalized over the whole gingiva. Usually, the symptoms appear during and after the eruption of permanent dentition. Gingival proliferation in HGF causes a variety of esthetic and practical issues. Depending on the size and intensity of the overgrowth, speech and chewing may be impaired. Moreover, diastema and prolonged primary dentition retention may occur.

**Case Reports:** This article describes the identification, management, and treatment approaches of four cases affecting a Syrian family who lived in Arbat refugee camp in Sulaymaniyah, Kurdistan, Iraq.

**Conclusion:** Proliferative fibrous outgrowth of the gingival tissue, with different degrees of involvement, is a hallmark of HGF. Surgery is frequently necessary to restore function and appearance, though varying degrees of recurrence is anticipated. Nonetheless, the psychological advantages of cosmetic improvement exceed the dangers of recurrence by a wide margin, especially in teenagers.

## 1. Introduction

Hereditary gingival fibromatosis (HGF) is an oral condition characterized by a slow and progressive enlargement of both attached and marginal gingiva. Synonyms for HGF include idiopathic gingival fibromatosis, elephantiasis gingivae, and fibromatosis gingivae. HGF is a rare condition with a prevalence of one per 175,000 populations, and its occurrence is equally distributed between the sexes. The condition typically begins just as the permanent teeth begin to erupt. Usually, it is transmitted as an autosomal dominant trait but recessive patterns of inheritance are also reported [1]. HGF can manifest alone or as a part of a syndrome in combination with other symptoms [2, 3].

Usually, the symptoms appear during and after the eruption of permanent dentition [4]. Since HGF does not affect edentulous ridges and fades or recedes after tooth extractions [5], it suggests that teeth are necessary for HGF to occur. Furthermore, gingival growth becomes worse during puberty, perhaps as a result of the increasing sex hormones. Typically, the hyperplastic gingiva is normal in color and consistency and the tendency of bleeding is minimal. In addition to having a nodular look, it has extensive gingival stippling [6]. The clinical manifestation of HGF is extremely variable. Both the mandible and the maxilla can have gingival overgrowth on the buccal and lingual surfaces. The swelling may be limited to a particular location or generalized over the whole gingiva. The most common regions for HGF are the maxillary tuberosities, the buccal gingiva around the mandibular molars, and the anterior part of the mandible [7].

For the affected cases, gingival enlargement in HGF causes a variety of aesthetic and practical issues. Depending on the size and intensity of the overgrowth, speech and chewing may be impaired. Moreover, diastema, prolonged primary dentition retention, and delayed eruption of permanent dentition have all been documented in the literature [5, 7]. In cases of significant gingival overgrowth, open bite, open lip posture, prominent lips, and an inability to approximate the lips are typical. Although HGF does not directly affect the alveolar bone, the enlarged gingiva may lead to more plaque buildup and poor plaque control, which can result in periodontitis, bone loss, and foul breath [3, 6].

Several gene mutations have been proposed as causative factors of HGF, such as SOS1 [7] and REST [8]. Recently, ALK and CD36 have also been mentioned as new candidate genes [9].

A literature search on PubMed showed only three case series on HGF. Hence, the present article is the fourth case series on the condition [10–12].

## 2. Case Presentation

This article describes the identification, management, and treatment approaches of four cases affecting a Syrian family who lived in Arbat refugee camp in Sulaymaniyah, Kurdistan, Iraq, presented to the periodontics department in Shorsh Dental Teaching Center.

### 2.1. Case 1

**Figure 1:**
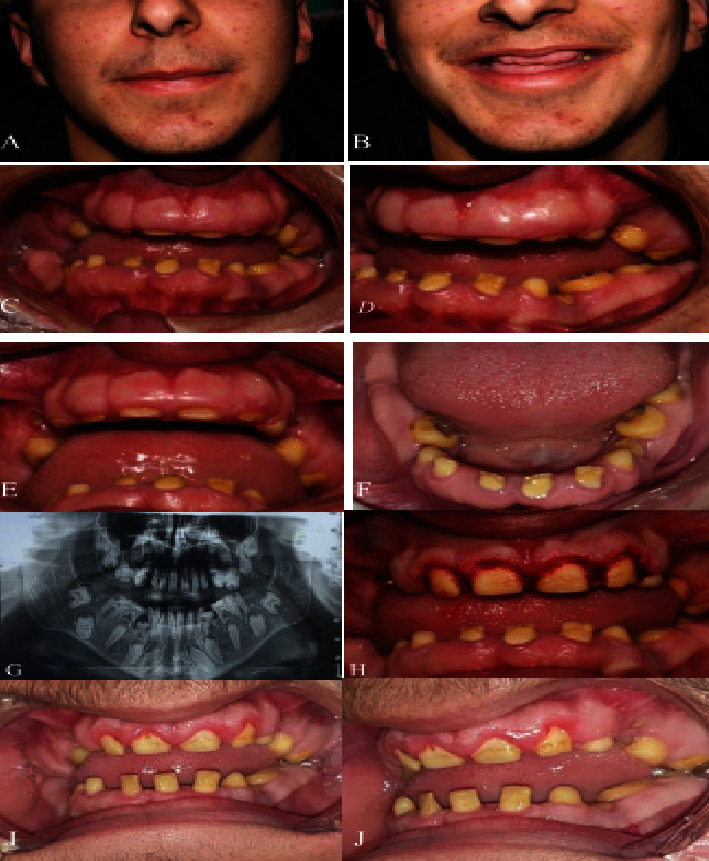
Case 1. (A) Extraoral frontal view reveals lip closure with tension. (B) Extraoral smile view. (C, D) Intraoral front and lateral views showing the gingival enlargement covering almost the entire teeth; there is severe open bite, spacing, and calculus formation around the teeth. (E) Close-up view of the upper anterior teeth shows dense, fibrous, and nodular gingiva. (F) Occlusal view of the lower arch shows retained deciduous and unerupted permanent teeth. (G) Panoramic radiograph shows the impacted canine, premolars, and second molar. (H) Gingivectomy for the upper incisor teeth. (I, J) Follow-up after 1 month shows a moderate improvement of the incisor teeth but with a high tendency of recurrence.

The eldest son of the family was a 16-year-old male complaining of excessive gingival appearance. He was fit and had no any systemic disease and no history of taking drugs. He gave a positive family history of the same condition shared with his father, brother, and sister. His weight and height were in normal limits without any obvious deformities in his body. His profile showed incompetent lips and muscle tension on mouth closing. During smiling a thick gingival biotype appeared that covered all the upper anterior teeth crowns except for a small portion of the incisal edge. Intraoral examination revealed several unerupted and semierupted teeth that were covered all-around by a thick, bulbous, pinkish, stippled, and severely enlarged gingiva that was firm on palpation on both maxillary and mandibular arches. This caused difficulty in eating and speaking and esthetic problems. There was generalized spacing between the teeth which were covered by calculus and plaque. Panoramic radiograph showed the impacted canine, premolars, and second and third molars with remaining deciduous molar teeth in all quadrants. No significant change in the alveolar bone was observed. The case was diagnosed as HGF (Figure 1).

Periodontal assessment revealed the presence of deep pockets (pseudo pocket up to 8 mm in the upper anterior teeth) and moderate calculus deposits on the lower anterior teeth with bleeding on probing in certain areas. The overall plaque index was 2.1, and the bleeding index was 1.7 in a way the lower teeth were worse.

Based on the evidence from the literature and the chief complaint of Case 1, the authors decided to perform gingivectomy. Written consent is taken from the patient and his family. The treatment plan was gingivectomy for the upper and lower anterior teeth. Because of the patient's status and demand for his gummy smile, the first gingivectomy of the upper anterior teeth was performed then scaling of the teeth. The patient refused to continue the treatment plan, and gingivectomy of lower teeth was not performed.

Due of the patient's status in the first appointment, gingivectomy for upper anterior teeth had been performed without gingivoplasty. After infiltration with lidocaine 2% and epinephrine 1:100,000 for centrals and laterals, the gingival pseudo pocket was measured and marked by the periodontal probe and 3 mm of the attached gingiva was preserved. An external bevel incision with blade no. 15 was done, and by using a sickle scaler, the tissue was removed. The area was rinsed and covered with wet gauze without using periodontal dressings. The patient was instructed to withdraw brushing of the upper anterior teeth for the first week. Mefenamic acid 500 mg tid was prescribed as postoperative pain management. The patient used chlorhexidine 0.12% gingival mouthwash for 2 weeks after the 1st week of the surgery.

### 2.2. Case 2

**Figure 2:**
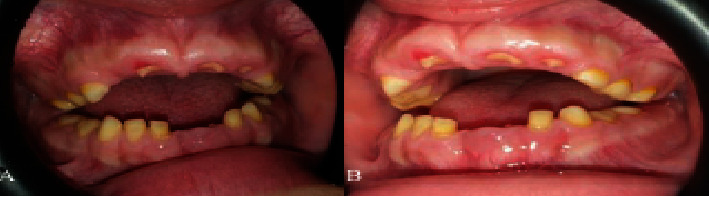
Case 2. (A, B) Front and lateral view photographs of the 11-year-old girl showing eruption failure of the lower central incisors and the upper anterior teeth are almost covered by gingival fibromatosis.

The second child of the same family was an 11-years-old girl presented with the same condition. She had no systemic disease, mental retardation, or medication history. Intraoral examination showed severe gingival enlargement that caused retention of most of the deciduous teeth. The gingiva was pinkish in color, firm, and has a nodular appearance. The patient had a severe open bite. Lower central incisors were exfoliated and upper central and lateral teeth just pierced the gingiva and a small portion of the crown appeared. No significant change in the alveolar bone was observed (Figure 2).

### 2.3. Case 3

**Figure 3:**
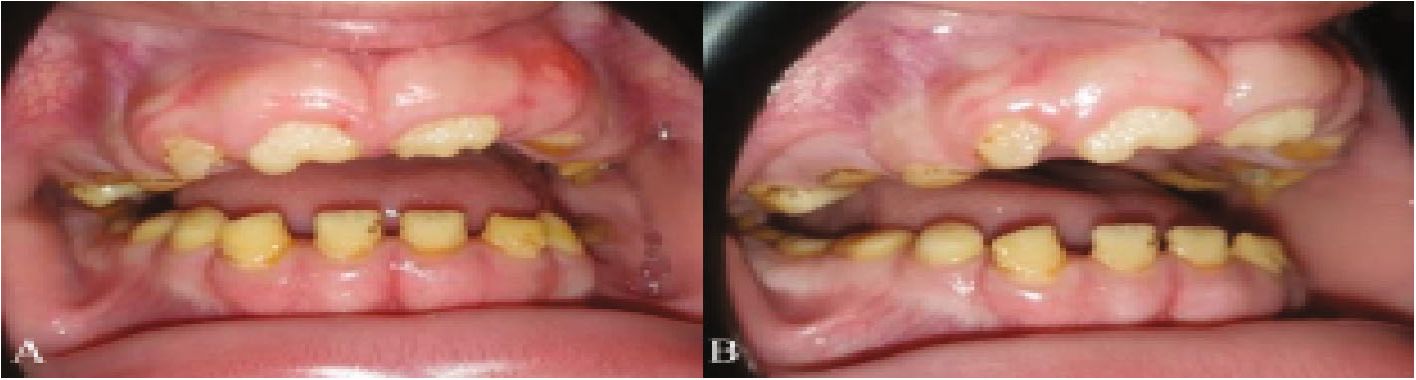
Case 3. (A, B) Front and lateral view photographs of the 9-year-old boy showing moderate gingival fibromatosis.

The third child of the same family who was a 9-year-old boy presented with the same condition. He had no systemic disease, mental retardation, and on medication. Intraoral examination showed severe gingival enlargement that caused retention of most of the deciduous teeth. The gingiva in the anterior teeth of both arches was pinkish in color, firm, and has a nodular appearance. The patient had a lateral open bite. Lower central and lateral incisors were erupted but covered by gingiva to the middle third of the teeth; the upper central and right lateral teeth erupted and only the incisal third of the teeth were visible. No significant change in the alveolar bone was observed (Figure 3).

### 2.4. Case 4

**Figure 4:**
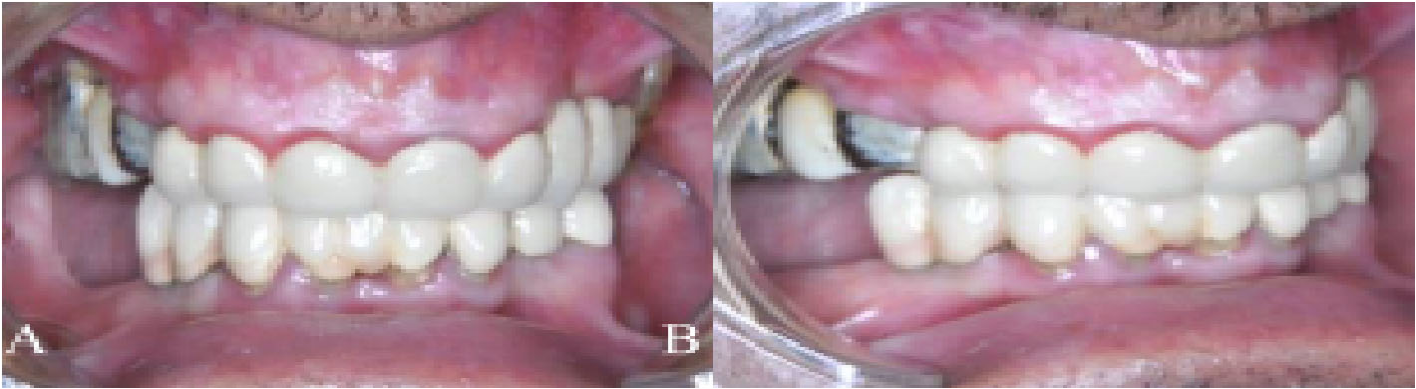
Case 4. (A, B) Front and lateral view photographs of the 48-year-old father showing a mild-to-moderate gingival fibromatosis with bridge prosthesis.

Their father was the last one of the family who attended for examination and was a 48-year-old man who complained of short clinical crowns. He had no systemic disease or mental retardation and was not on medication but he was a smoker. He was misdiagnosed and mistreated before with a fixed prosthetic appliance for his upper left premolar to right canine and a lower appliance from left premolar to right premolar. No significant change in the alveolar bone was observed (Figure 4). The patient had no complains and was not seeking any treatment.

## 3. Discussion

In this paper, we reported four members of a family with HGF which is a rare hereditary condition characterized by abnormal enlargement of the gingival tissue. The clinical manifestation of the reported family, suggested the HGF was apparently transmitted as an autosomal dominant trait with a high degree of penetrance, since without an exception, all the children had been transmitted the disorder from their father.

As stated before, HGF can happen as an isolated case or associated with syndromes. In the syndromic condition, the presence of mental retardation, hypertrichosis, and epilepsy are reported in the literature [13]. The presented family appears to be nonsyndromic as the members lack the aforementioned symptoms.

Differential diagnosis of gingival enlargements includes enlargement associated with hormonal changes (such as in pregnancy and puberty), various inflammations, drug-induced gingival hyperplasia, tumors, cysts, or enlargements due to genetic disorders [14]. In the present family report, the diagnosis of HGF is based on the family history and clinical examination.

In terms of severity of the condition, gingival hyperplasia was severe in Case 1, moderate in Cases 2 and 3, and mild in Case 4. The severity of Case 1 who was a 16-year-old teenage boy was in agreement with the previous reported cases, since HGF is known to worsen around the time of puberty due to hormonal changes.

There is no definite cure for HGF, rather it could be controlled with varying degrees of success. Not every case of HGF needs treatment; especially when the condition is minimal, it can be managed by deep scaling of the teeth, meticulous oral hygiene regimen, and regular follow-up. However, when the enlargement is severe enough to cause esthetic, functional, speech, and psychological problems, gingivectomy and gingivoplasty are required [3].

One month follow-up after the procedure for Case 1 showed a moderate improvement in the gingival overgrowth but with a clear tendency towards recurrence. Relapse is a common outcome in the treatment of HGF, and it is seen more in children and teenagers as compared to adults [15]. It appears that the majority of the time, maintaining good oral hygiene prevents the overgrowth from reoccurring. Yet, even with proper oral hygiene, recurrence of HGF is still possible due to a number of inherited predispositions [16]. Consequently, it is not possible to foresee how effectively a treatment will function over time. However, even for a temporary period, the psychological advantages of gingivectomy should not be underestimated for a teenager like our case.

Regarding the other members of the family, children of 9 and 11 years old were given special instruction and motivation on oral hygiene using the tell-show-do technique. The best moment to intervene, according to numerous authors, is after the permanent dentition has emerged because treatment during the mixed dentition increases the chances of a recurrence [13, 17]. As for the father, his gingival status was not as bad as his children, since most of the gingival enlargement has subsided in his age. Besides, he refused to get any treatment. Therefore, we advised him to do a better homecare for his inflamed gingiva especially with the presence of a lower and upper bridge on his anterior teeth.

To our knowledge and based on a literature search in PubMed database, the current report is the fourth family case series [10–12]. Out of these, only one article reported gingivectomy and a 1-month follow-up with a promising result [12]. The present case series have several points in common with the other published family case series on HGF; most importantly, the involvement of more than one generation, early onset in children, and exacerbation of symptoms during puberty time [10–12].

## 4. Conclusion

Proliferative fibrous outgrowth of the gingival tissue, with different degrees of involvement, is a hallmark of HGF. Surgery is frequently necessary to restore function and appearance, though varying degrees of recurrence is anticipated. Nonetheless, the psychological advantages of cosmetic improvement exceed the dangers of recurrence by a wide margin, especially in teenagers.

## Data Availability

The data used to support the findings of this study are available from the corresponding author upon request.
